# 55270 The Histone Methyltransferase SETDB2 Regulates Inflammation in Normal and Diabetic Wound Repair

**DOI:** 10.1017/cts.2021.441

**Published:** 2021-03-30

**Authors:** Aaron denDekker, Frank Davis, Andrew Kimball, Matthew Schaller, Amrita Joshi, Ronald Allen, Katherine Gallagher

**Affiliations:** 1 University of Michigan; 2 University of Alabama-Birmingham; 3 University of Florida

## Abstract

ABSTRACT IMPACT: Our data reveal a histone modifying enzyme involved in regulating inflammation that may be a novel target for treating non-healing diabetic wounds. OBJECTIVES/GOALS: We investigate molecular mechanisms that regulate the inflammatory phenotype of macrophages in normal and diabetic wound healing. Our goal is to identify novel pathways that may be used to better treat diabetic patients with non-healing wounds. METHODS/STUDY POPULATION: We utilize normal and transgenic murine models on standard chow or high-diet to identify chromatin modifying enzymes involved in regulating macrophage function during wound healing. We validate our murine studies with human blood monocytes or wound macrophages from diabetic patients undergoing limb amputation surgery. RESULTS/ANTICIPATED RESULTS: We have identified the histone methyltransferase SETDB2 as a regulator inflammation in normal and diabetic wound macrophages. We found that SETDB2 was dependent on IFNβ singaling and that both IFNβ and Setdb2 expression were impaired in diabetic wound macrophages. Further, we show that SETDB2 regulates inflammatory response and immune cell trafficking pathways. We also show that SETDB2 genomic localization is dependent on *NFÎºÎ’ deposition of the promoter. DISCUSSION/SIGNIFICANCE OF FINDINGS: Our results indicate that SETDB2 is a regulator of macrophage plasticity and that SETDB2 expression is impaired in diabetic wound macrophages leading to hyper-inflammatory response and delayed wound healing. These data provide a novel potential therapeutic pathway for treating non-healing diabetic wounds.

